# A Neurotoxic Phospholipase A_2_ Impairs Yeast Amphiphysin Activity and Reduces Endocytosis

**DOI:** 10.1371/journal.pone.0040931

**Published:** 2012-07-23

**Authors:** Mojca Mattiazzi, Yidi Sun, Heimo Wolinski, Andrej Bavdek, Toni Petan, Gregor Anderluh, Sepp D. Kohlwein, David G. Drubin, Igor Križaj, Uroš Petrovič

**Affiliations:** 1 Department of Molecular and Biomedical Sciences, Jožef Stefan Institute, Ljubljana, Slovenia; 2 Department of Molecular and Cell Biology, University of California, Berkeley, California, United States of America; 3 Institute of Molecular Biosciences, University of Graz, Graz, Austria; 4 Department of Biology, Biotechnical Faculty, University of Ljubljana, Ljubljana, Slovenia; Institut Curie, France

## Abstract

**Background:**

Presynaptically neurotoxic phospholipases A_2_ inhibit synaptic vesicle recycling through endocytosis.

**Principal Findings:**

Here we provide insight into the action of a presynaptically neurotoxic phospholipase A_2_ ammodytoxin A (AtxA) on clathrin-dependent endocytosis in budding yeast. AtxA caused changes in the dynamics of vesicle formation and scission from the plasma membrane in a phospholipase activity dependent manner. Our data, based on synthetic dosage lethality screen and the analysis of the dynamics of sites of endocytosis, indicate that AtxA impairs the activity of amphiphysin.

**Conclusions:**

We identified amphiphysin and endocytosis as the target of AtxA intracellular activity. We propose that AtxA reduces endocytosis following a mechanism of action which includes both a specific protein–protein interaction and enzymatic activity, and which is applicable to yeast and mammalian cells. Knowing how neurotoxic phospholipases A_2_ work can open new ways to regulate endocytosis.

## Introduction

Phospholipases A_2_ (PLA_2_s) are hydrolytic enzymes that catalyze the cleavage of the ester bond at the *sn-2* position of glycerophospholipids [1]. PLA_2_s change the lipid composition of membranes, are key enzymes at regulating the production of active lipid mediators (*e.g.* arachidonic acid), and at the same time, through generation of lysophospholipids, they affect membrane curvature [2]–[4]. Members of the family of secreted phospholipases A_2_ (sPLA_2_s) are present in a variety of mammalian tissues as well as in venoms of different animals [5], [6]. Snake venom sPLA_2_s display a wide range of pharmacological activities, including presynaptic neurotoxicity [5]. Presynaptically neurotoxic sPLA_2_s affect the nerve terminals of motor neurons by blocking signal transmission over the synaptic cleft [7] and cause characteristic morphological changes in the affected nerve terminal, *e.g.* swollen and damaged mitochondria, Ω-shaped invaginations at the plasma membrane coated with electron-dense material (presumably clathrin), formation of large vesicles, and reduced synaptic vesicle number [8]–[11].

The primary target of the toxins is the motor nerve terminal, but there is no agreement on either the precise site of action of the toxins or their mechanism of action. It was proposed that presynaptic neurotoxicity is a result of solely their extracellular phospholipase activity. The sPLA_2_ neurotoxins have been suggested to stimulate synaptic vesicle fusion with the presynaptic membrane and on the other hand prevent their recycling, by formation of inverted-cone-shaped lysophospholipids and cone-shaped unsaturated free fatty acids [11], [12]. Alternatively, both extra-and intracellular activities, involving PLA_2_ enzymatic activity as well as physical interactions with specific intracellular proteins, have been proposed [7].

Ammodytoxins are presynaptically neurotoxic sPLA_2_s from the venom of the nose-horned viper (*Vipera ammodytes ammodytes*) and they inhibit the release of the neurotransmitter acetylcholine from motor neurons [13]. The active form of ammodytoxin A (AtxA) has been expressed in the budding yeast *Saccharomyces cerevisiae* where it has been shown not to be cytotoxic, although enzymatically active and localized throughout the cytosol, and to inhibit G_2_ cell-cycle arrest [14], [15]. Extracellularly added AtxA has no such effects on yeast cells. Since AtxA interacts with evolutionarily highly conserved proteins, such as calmodulin and 14-3-3 proteins [16], [17], the intracellular mechanism of action of AtxA is expected to be conserved among eukaryotic cells as well. In this study, our primary objective was to find the targets of AtxA activity in a genome-wide approach, and to more closely investigate the effects of AtxA on endocytosis which was identified as a targeted biological process in the screen. Specifically, we studied the effect of AtxA in the context of its genetic and functional interaction with Rvs161 and Rvs167 proteins, which are yeast homologs of mammalian amphiphysin and are involved in the scission of the newly formed endocytic vesicle from the plasma membrane [18].

## Results

### AtxA is in Genetic Interaction with Genes Involved in Endocytosis

We expressed AtxA in all viable gene deletion mutant strains of *S. cerevisiae* and performed a synthetic dosage lethality (SDL) screen. We determined the growth phenotype of all the obtained strains, and those with a significantly inhibited growth were selected as carriers of deletions of genes in genetic interaction with AtxA. Four hundred twelve genes were identified in the screen. A recent genome-wide study has identified 400 genes required for internalization of plasma membrane proteins [19]. Of these, 47 were found in our SDL screen, representing a significant degree of enrichment (p-value 0.007, hypergeometric distribution). Analysis based on gene ontology annotations of the SDL hits showed that AtxA is in genetic interaction with 48 genes (of which only 9 overlap with the above mentioned 47) involved in endocytosis, actin cytoskeleton organization and vesicle-mediated transport. The endocytosis related genes identified at the highest confidence level were *RVS161*, *YAP1802*, *LDB19*, *VPS1*, *APP1* and *BMH1*, and their genetic interactions were confirmed by growth curve assay. Notably, *RVS161*, *YAP1802* and *BMH1* have paralogs in the yeast genome (*RVS167*, *YAP1801* and *BMH2*, respectively) which however do not genetically interact with AtxA, as established by the growth curve assay (data not shown). One possible explanation of the difference between the mutants of paralogs is that the amount of active AtxA only suffices for functional titration of the minor isoform molecules available in the cell after the deletion of the major isoform gene, but not vice versa. This can explain the difference between the pairs of *BMH1/2* and *YAP1801/1802* genes that code for highly similar (96% and 44% amino acid sequence identity, respectively) and functionally interchangeable proteins which differ in their cellular abundance between three-fold and ten-fold from their respective isoform [20]–[23]. According to this explanation, AtxA inhibits the activity of minor isoforms Bmh2 and Yap1801 proteins when they are the sole isoform present in the cell to the level sufficient to inhibit the growth. Based on high structural and functional similarity between Bmh1/2 and Yap1801/1802 it is then reasonable to assume that AtxA also partially inhibits the activity of Bmh1 and Yap1802, however this inhibition does not affect the growth rate. On the other hand, in the case of *RVS161/167* genes, which are less similar (24% amino acid sequence identity), the absence of the approximately two-fold less abundant Rvs161 isoform [23] resulted in growth defect in the presence of AtxA. To establish the reason for the specific genetic interaction with *RVS161*, we decided to investigate in more detail the molecular interplay between AtxA and Rvs161/167 proteins.

### AtxA Reduces Endocytosis in Yeast

In mammals, neurotoxic sPLA_2_s inhibit endocytosis, which is one of the biological processes in which Rvs161 and Rvs167 in yeast have an important role. To determine whether AtxA expression in yeast caused defects in uptake or subsequent delivery of endocytic contents, we measured the uptake rate of a fluid-phase endocytosis marker, the membrane-impermeant fluorescent dye Lucifer Yellow (LY) [24], in the strains with either integrated or plasmid-borne AtxA gene. In the cells with a single copy of integrated AtxA gene, the relative uptake rate of LY was 0.59±0.07 of the control strain, whereas in the cells with multi-copy AtxA genes on plasmids the relative uptake rate was 0.38±0.08 of the control strain. AtxA was thus found to reduce endocytosis in yeast.

### AtxA does not Affect the Number of Endocytic Sites in Yeast Cells

We hypothesized that AtxA affects endocytosis by reducing the number of endocytic sites present in a cell, and/or by affecting the temporal and/or spatial dynamics of vesicle formation itself. To test whether AtxA affects the number of endocytic sites in the cell, we determined the average number of endocytic sites per cell in a strain expressing AtxA from a plasmid. To this end, confocal microscopy and a green fluorescent protein (GFP)-tagged clathrin coat protein Sla1, which is an appropriate marker to investigate early steps in endocytosis [18], [25], were used. As determined by manual counting, the average number of endocytic sites per wild-type cell (defined as the number of Sla1-GFP patches), was 21.4±7.3. No significant change was observed in the strain expressing AtxA (21.6±9.1). The average patch numbers were similar when the automatic counting was used: 17.9±2.5 in the control strain and 17.3±2.4 in the AtxA-expressing strain. The numbers observed using the automated counting are slightly lower because the algorithm used can not entirely distinguish between two structures that are in very close proximity or are partially overlapping. The overall conclusion from these experiments is that the number of endocytic sites is not affected by AtxA, indicating that the alteration of the spatial and/or temporal dynamics of endocytic proteins is more likely the mode of action of AtxA for the reduction of endocytosis in yeast cells.

### AtxA Alters the Spatial and Temporal Dynamics of Clathrin-dependent Endocytosis

**Figure 1:**
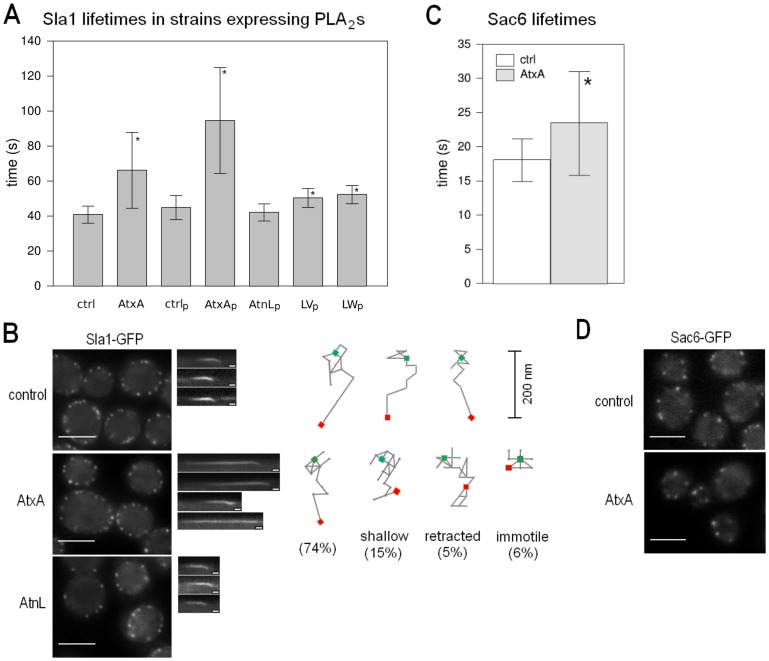
AtxA affects the dynamics of endocytic vesicle formation. **A** Average Sla1-GFP patch lifetimes in the wild-type, and cells expressing AtxA, AtnL, and the LV and LW mutants of AtnL. **B** Analysis of Sla1-GFP dynamics. In the left panels, localizations of Sla1-GFP in the wild-type, AtxA-expressing and AtnL-expressing strains are shown. In the middle panels, representative kymographs are shown. For the AtxA-expressing strain, kymographs for a normally internalized, shallow internalized, retracted and immotile patch are shown. All kymographs are oriented such that the cell interior faces downwards. In the right panels the trackings of three Sla1-GFP patches from the wild-type cells and four patches from AtxA-expressing cells representing one normally internalized, shallow internalized, retracted and immotile patch each, are shown. Noted is also the percentage of their occurrence in the population. For each frame of the movie, the center of the patch was determined. Green rectangles denote the initial position and red rectangles the final position of the patches. Consecutive positions are connected with lines. All traces are oriented with the cell interior facing down. **C** Average patch lifetime of Sac6-GFP in a strain expressing AtxA from plasmid and corresponding control strain. **D** Localization of Sac6-GFP in a strain expressing AtxA from plasmid and corresponding control cells. Ctrl – control strain for the integrated AtxA, AtxA – strain expressing AtxA from singe copy integrated in the genome, ctrl_p_ – control strain with empty plasmid, AtxA_p_, AtnL_p_, LV_p_ and LW_p_ – strains expressing the respective proteins from plasmid clones. To determine the average patch lifetimes at least 100 patches from several cells were analyzed unless otherwise noted. The bars on graphs mark the standard deviation. Sla1-GFP movies were taken with a 1 frame/second interval and Sac6-GFP movies with a 4 frames/second interval. Scale bar on the micrographs is 4 µm and on the kymographs 10 seconds. sPLA_2_-expressing strains were compared to the corresponding control strains, and p-values were calculated using the *t-*test at a 95% confidence interval. Statistically significant differences are denoted with a star (*).

To test whether AtxA affects the spatial and temporal dynamics of endocytosis, we used real-time fluorescence microscopy to analyze the behavior of Sla1-GFP in the wild-type strain expressing AtxA either from a plasmid or after chromosomal integration. The average lifetime of the Sla1-GFP patches in the strain expressing AtxA from a single copy integrated in the genome was significantly increased by 1.67-fold (Figure 1A). When AtxA was expressed from a plasmid, the average lifetime increase was 2.1-fold (Figure 1A). The longer before inward movement part of the lifetime of Sla1 is due to a delay in the efficient start of the scission step [18], causing Sla1 to be stalled at the plasma membrane, waiting for the later arriving proteins to perform their function and complete scission. A similar increase in the before inward movement part of the lifetime of Sla1 has also been shown in *rvs161Δ* and *rvs167Δ* strains [18].

To determine whether the enzymatic activity of AtxA is the cause for the observed endocytic defect, we analyzed also the behavior of the Sla1-GFP patches in the wild-type strain expressing from a plasmid an enzymatically inactive homolog of AtxA, ammodytin L (AtnL) [26]. Here, the average Sla1-GFP patch lifetime did not increase (Figure 1A). We next expressed two AtnL mutants with restored enzymatic activity [27] and observed a relatively small but significant increase in the average lifetime of Sla1-GFP patches (Figure 1A): the LW mutant had a greater effect, with 1.24-fold increase in the average lifetime and significantly higher number of patches with lifetimes longer than 55 seconds (Supporting Information S1), than the LV mutant (1.20-fold increase). The effects of the mutants correlate well with their enzymatic activity on PC-rich phospholipid vesicles [27]. These results strongly indicate that the observed effect on endocytosis is the consequence of the PLA_2_ enzymatic activity. We next analyzed Sla1-GFP patch dynamics in the AtxA-expressing strain and found that approximately 6% of the Sla1-GFP patches were not internalized (Figure 1B), whereas in the control strain virtually all of the patches were internalized. Moreover, 15% of the internalized patches appeared to have a more shallow internalization path when AtxA was expressed (Figure 1B), which was not observed in the control strain. The failure in internalization of the 6% of the Sla1-GFP patches could be due to a partial effect of AtxA on the actin cytoskeleton, especially since it has previously been shown that AtxA affects the actin cytoskeleton in mammalian cells [28]. We thus analyzed the effect of AtxA expression on the yeast fimbrin Sac6, an actin-bundling protein [29]. In the *sac6Δ* strain, the inward movement of endocytic vesicles is abolished [18]. In the AtxA-expressing strain, Sac6-GFP patch lifetimes increased by 1.3-fold (Figure 1C) while no difference was observed in the localization of the protein (Figure 1D).

### Rvs161 is Needed for the Effect of AtxA on Endocytosis

**Figure 2:**
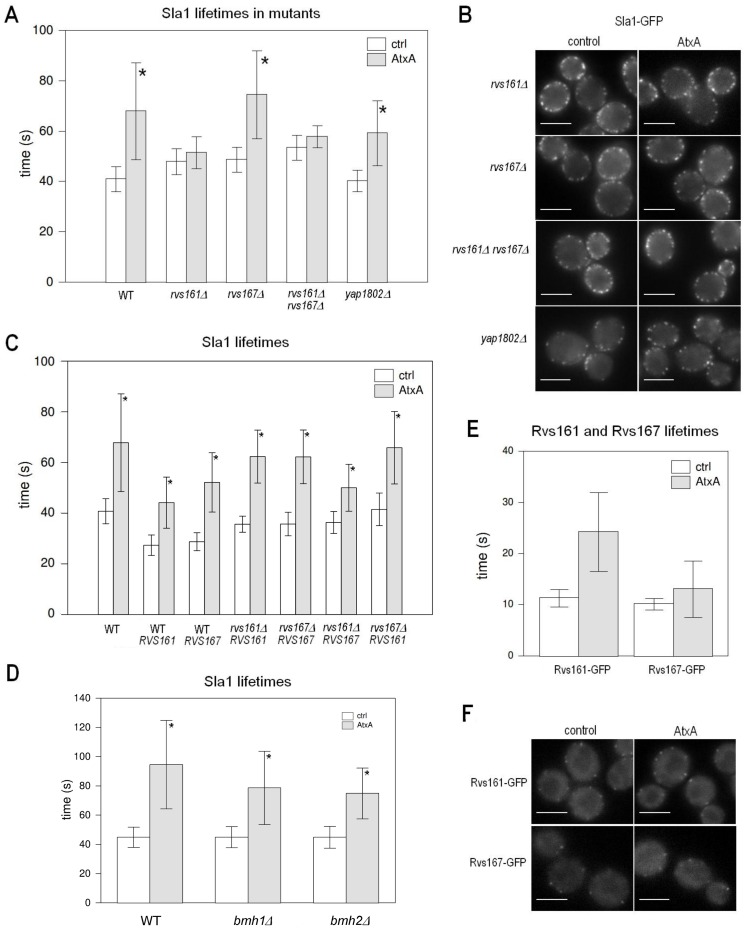
Rvs proteins are needed for the effect of AtxA on endocytosis. **A** Average Sla1-GFP patch lifetimes for different *rvs* deletion strains and *yap1802Δ* deletion strain expressing AtxA from a single copy integrated in the genome, and corresponding control strains. **B** Localization of Sla1-GFP in *rvs161Δ*, *rvs167Δ*, the *rvs161Δ rvs167Δ* double deletion strain and *yap1802Δ*. **C** Effect of additional expression of either *RVS161* or *RVS167* on average Sla1-GFP patch lifetimes for WT and *rvs* deletion strains expressing AtxA from a single copy integrated in the genome and corresponding control strains. **D** Average Sla1-GFP patch lifetimes for the wild-type and *bmh1Δ* and *bmh2Δ* deletion strains expressing AtxA from plasmid, and corresponding control strains. **E** Average lifetimes of Rvs161-GFP (left) and Rvs167-GFP (right) patches for the wild-type (ctrl) and AtxA-expressing cells. **F** Localization of Rvs161-GFP and Rvs167-GFP in AtxA-expressing and control cells. The bars on graphs mark the standard deviation. To determine the average patch lifetimes at least 100 patches from several cells were analyzed. Sla1-GFP movies were taken with a 1 frame/second and Rvs161-GFP and Rvs167-GFP movies with a 4 frames/second interval. Scale bar on the micrographs is 4 µm. AtxA-expressing strains were compared to the corresponding control strains, and p-values were calculated using the *t-*test at a 95% confidence interval. Statistically significant differences are denoted with a star (*).

Rvs161 and Rvs167 are amphiphysin homologs [30], involved in invagination and scission of the newly formed vesicle from the plasma membrane [18], [31]. They contain an N-BAR (Bin/Amphiphysin/Rvs) domain that senses and induces membrane curvature. To test whether the roles of Rvs161/167 in endocytosis could explain the specific genetic interaction of AtxA with *RVS161*, we analyzed the effect of AtxA on Sla1-GFP patches in different *rvs* deletion backgrounds. Upon AtxA expression in the *rvs161Δ* strain the average Sla1-GFP patch lifetime did not increase significantly (Figure 2A). On the contrary, in the *rvs167Δ* strain the average Sla1-GFP patch lifetime upon AtxA expression was significantly (by 1.53-fold) increased (Figure 2A), which is somewhat less than the increase in the average Sla1-GFP patch lifetime after AtxA expression in the wild-type strain. In the *rvs161Δ rvs167Δ* double deletion strain, same as in the *rvs161Δ* single deletion strain, AtxA expression had no significant effect on the average Sla1-GFP patch lifetime (Figure 2A). In all the *rvs* deletion mutants, AtxA had no observable effect on the localization of Sla1-GFP (Figure 2B).

To test whether the presence of Rvs161, but not Rvs167, is necessary for AtxA to affect the dynamics of endocytosis, or alternatively if the effect of deletion of one amphiphysin isoform on the effect of AtxA could be rescued by the other isoform, we analyzed the effect of AtxA on the Sla1-GFP patch lifetime in the strains with all possible combinations of deletion and additional expression of *RVS161/167* genes. Additional expression of Rvs161 as well as Rvs167 decreased the Sla1-GFP patch lifetime by ∼1.45-fold in the wild-type background (Figure 2C). When AtxA was expressed in these strains, it increased the Sla1-GFP patch lifetime to a similar extent as in the wild-type strain (by 1.61-fold in the strain additionally expressing *RVS161* and by 1.81-fold in the strain additionally expressing *RVS167*) (Figure 2C), indicating that AtxA and amphiphysin isoforms have opposing activities, and that the amount of Rvs161 or Rvs167 in the wild-type strain does not limit the extent of reduction of endocytosis by AtxA. As expected, additional expression of Rvs161 in the *rvs161Δ* strain and of Rvs167 in the *rvs167Δ* strain resulted in an increase in the Sla1-GFP patch lifetime following AtxA expression comparable to that observed in the wild-type strain (Figure 2C). Comparison of Sla1-GFP patch lifetimes in the absence of AtxA expression revealed that strains over-expressing Rvs161 and Rvs167 in these corresponding deletion mutants had a stronger effect decreasing the lifetime than in the strains where the homolog was deleted (Figure 2C). That is, additional expression of Rvs167 in the *rvs161Δ* strain and of Rvs161 in the *rvs167Δ* strain decreased the lifetime by only 1.29-and 1.11-fold, respectively, compared to ∼1.35-fold in the corresponding gene deletion strains (Figure 2C), demonstrating that the two isoforms cannot completely functionally complement each other. This is in agreement with the notion that in endocytosis Rvs161 and Rvs167 function as a heterodimer [32], [33]. The difference between the observed decreases in Sla1-GFP patch lifetimes between the *rvs161Δ* strain additionally expressing Rvs167 and the *rvs167Δ* strain additionally expressing Rvs161 can be attributed to the expected approximately two-fold higher abundance of Rvs167 compared to Rvs161 when expressed from their native promoters [23], which were used in these experiments. Expression of AtxA increased the Sla1-GFP patch lifetime in the *rvs167Δ* strain almost to the same extent as in the wild-type strain, regardless of whether Rvs161 was additionally expressed or not: by 1.53-fold in the strain where Rvs161 was not additionally expressed (Figure 2A), and by 1.59-fold in the *rvs167Δ* strain where Rvs161 was additionally expressed (Figure 2C). Contrary to this, in the *rvs161Δ* strain, where AtxA did not increase the Sla1-GFP patch lifetime (Figure 2A), upon additional expression of the Rvs167 protein AtxA increased the lifetime by only 1.38-fold (Figure 2C). Thus, effect of AtxA on endocytosis occurs also in the absence of Rvs161, but only to a lesser extent than in its presence, and only if additional Rvs167 is present, indicating that AtxA activity counteracts the activity of yeast amphiphysin homologs, especially of Rvs161.

Based on the genetic interaction of AtxA with *YAP1802* we proposed that AtxA significantly inhibits the yeast AP180 homolog Yap1801 (see above). Similarly as Rvs161/Rvs167 proteins, Yap1801/1802 proteins play an important role in endocytosis and we therefore tested also the effect of AtxA in the *yap1802Δ* deletion strain where endocytosis relies only on Yap1801. The average Sla1-GFP patch lifetime in the *yap1802Δ* deletion strain was not significantly different from that in the wild-type strain (Figure 2A), indicating functional complementation between Yap1801 and Yap1802. After AtxA expression in the *yap1802Δ* deletion strain, the lifetime increased by 1.47-fold (Figure 2A), which is somewhat less than in the wild-type background (1.67-fold) indicating a partial inhibition of the role of Yap1801 in endocytosis by AtxA, but less pronounced than in the case of Rvs161.

In the SDL screen we found *BMH1* in genetic interaction with AtxA and reasoned that AtxA significantly inhibits the activity of Bmh2 (see above). *BMH1* and *BMH2* encode highly conserved 14-3-3 proteins that function as adaptors in a wide range of cellular processes [34]. At least Bmh2 is likely involved in endocytosis since it has been identified as a suppressor of the clathrin heavy chain gene deletion [35] and is in negative genetic interaction with *SLA1* as well as physically interacts with Yap1801 [36], [37]. To address functional relation between AtxA and 14-3-3 proteins in the context of inhibition of endocytosis, we measured the Sla1-GFP patch lifetime in *bmh1Δ* and *bmh2Δ* deletion strains (Figure 2D). The lifetime in the deletion strains without AtxA was indistinguishable from that in the wild-type strain. Expression of AtxA from the multi-copy plasmid in the deletion strains prolonged the Sla1-GFP lifetime by 1.75-fold in the *bmh1Δ* and by 1.67-fold in the *bmh2Δ* strain (Figure 2D). The effect of AtxA was therefore diminished compared to the wild-type strain (2.1-fold increase in the lifetime after plasmid-based AtxA expression), and the extent of this reduction was similar to the one observed in the *yap1802Δ* deletion strain (1.71-fold increase in the lifetime after plasmid-based AtxA expression), indicating that both 14-3-3 and AP180 proteins are involved in the reduction of endocytosis by AtxA in yeast cells.

To further explore the observed difference between the effects of AtxA on Rvs161 and Rvs167, we examined the effect of AtxA expression on the lifetimes of the Rvs161-GFP and Rvs167-GFP patches. AtxA expression doubled the average Rvs161-GFP patch lifetime, whereas it had a much smaller effect on Rvs167-GFP patch lifetime (Figure 2E). These data further indicate that inhibition of the function of Rvs161, rather than Rvs167, is part of the mechanism of AtxA to affect the dynamics of endocytosis, in agreement with the difference in the effect of AtxA in different *rvs* deletion/additional expression strains, as described above.

To test whether AtxA functions by completely inhibiting the activity of Rvs161 and thus mimicking its deletion, we analyzed the internalization profile of Sla1-GFP patches. In the AtxA-expressing strain, among the patches that internalize (*i.e.* 94% patches), approximately 5% retract after the initial internalization (example shown in Figure 1B), compared to the control strain where we determined that the percentage of retracted vesicles is below 1%, in accordance also with data in the literature [18]. The increase in the percentage of retracted vesicles caused by AtxA is still much lower than that determined for the *rvs161Δ* and *rvs167Δ* single deletion strains (26% and 29%, respectively) and the *rvs161Δ rvs167Δ* double deletion strain (24%). Expression of AtxA increased the percentage of retracting vesicles by approximately 5% also in *rvs* deletion strains: to 31% in *rvs161Δ*, 34% in *rvs167Δ* and 27% in *rvs161Δ rvs167Δ*. This result indicates that the effect of AtxA does not mimic amphiphysin deletion, but rather that AtxA inhibits a specific function of Rvs161. In accordance with this notion, we observed no differences in the localization of Rvs161-GFP and Rvs167-GFP in the AtxA-expressing strain compared to the localization in the wild-type background (Figure 2F).

**Figure 3:**
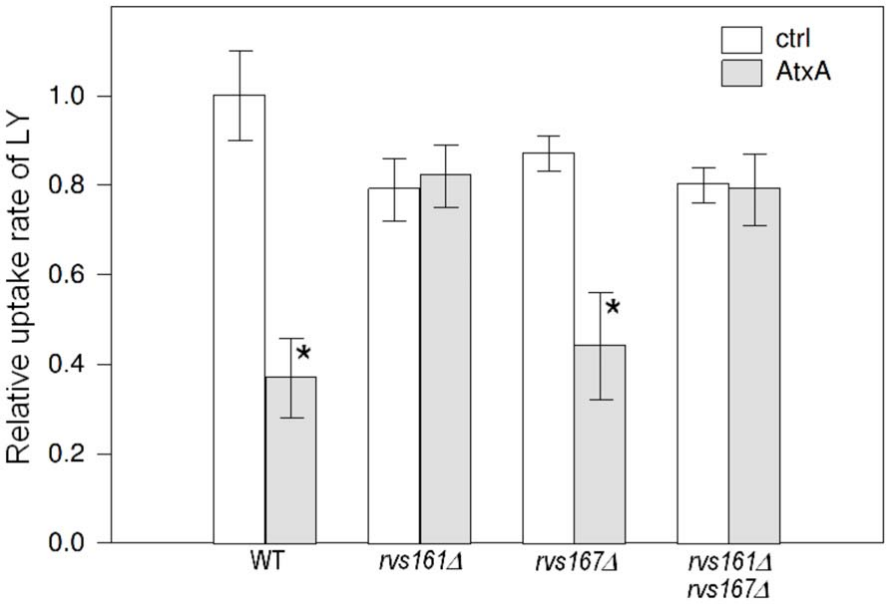
AtxA inhibits endocytosis in an Rvs161-dependent manner. Relative uptake rates of LY in AtxA-expressing and control strains with wild-type and different *rvs* deletion backgrounds. The bars represent standard deviations of at least three independent measurements. AtxA-expressing strains were compared to the corresponding control strains, and p-values were calculated using the *t-*test at a 95% confidence interval. Statistically significant differences are denoted with a star (*).

To test the effect of AtxA in different *rvs* deletion backgrounds with a different method, we next determined the uptake rate of LY in these strains (Figure 3). The uptake rate of LY in the *rvs161*Δ strain that also expressed AtxA was comparable to the uptake rate in the control *rvs161*Δ strain, which again indicates that the presence of Rvs161 is needed for the effect of AtxA on endocytosis. Moreover, in the *rvs167Δ* background, the uptake rate of LY was significantly decreased after AtxA expression and the extent of this effect was similar to the reduction observed in the wild-type strain. In the *rvs161Δ rvs167Δ* double deletion, the uptake rate of LY did not change significantly after AtxA expression (Figure 3). These results provided further proof that the function of Rvs161 rather than Rvs167 is the primary target of AtxA for the reduction of endocytosis.

### Enzymatic Activity of AtxA is Membrane Curvature Dependent

**Figure 4:**
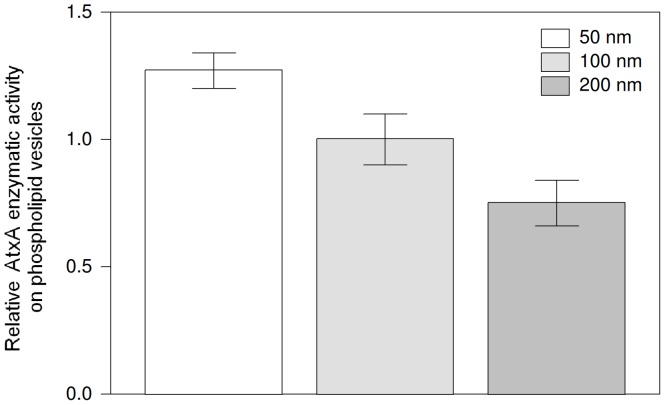
AtxA enzymatic activity is membrane curvature dependent. Relative enzymatic activity of AtxA on phospholipid vesicles of different diameters (50 nm, 100 nm and 200 nm) is shown. The measurements were normalized to the phospholipolytic activity on 100 nm vesicles. The bars represent standard deviation of three independent measurements. The differences are statistically significant based on the unpaired t-test at 95% confidence interval.

In endocytosis, Rvs161 exerts its activity on a highly curved vesicle membrane [18]. It has previously been shown that sPLA_2_s have higher enzymatic activity on substrates present in non-planar membranes [38], [39]. To determine whether the activity of AtxA is also membrane curvature dependent, we determined its relative PLA_2_ enzymatic activity on phospholipid vesicles of different sizes by monitoring displacement of a fluorescent fatty acid analogue from the fatty acid-binding protein. The enzymatic activity was measured on vesicles of 50 nm, 100 nm and 200 nm nominal diameters and the results normalized to the activity on the 100 nm vesicles. The relative enzymatic activity on the 50 nm vesicles was approximately 27% higher, and on the 200 nm vesicles 25% lower, than on the 100 nm vesicles (Figure 4). The differences were relatively small, but statistically significant. This shows that AtxA has a preference for hydrolysis of phospholipids within vesicles of smaller diameter that have higher membrane curvature, which is comparable to those of the endocytic and synaptic vesicles. Due to their instability, the enzymatic activity on 30 nm vesicles could not be tested (Supporting Information S1).

### Yeast 14-3-3 Proteins, Bmh1 and Bmh2, Bind to Several Toxic sPLA_2_s and Phospholipid Vesicles

**Figure 5:**
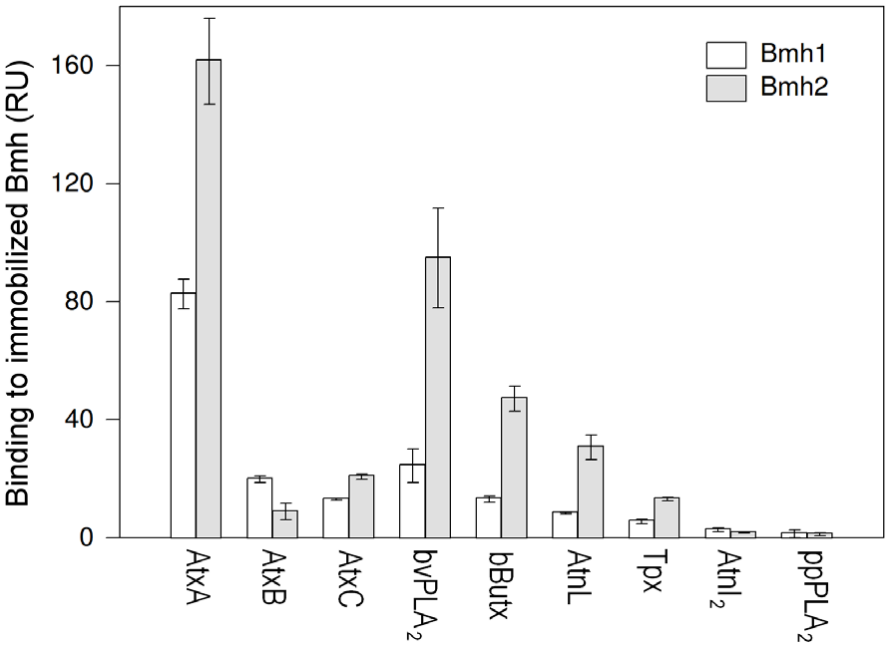
Several neurotoxic sPLA_2_s bind to yeast 14-3-3 proteins. All tested neurotoxic sPLA_2_s as well as AtnL bind to both Bmh1 and Bmh2. No binding was detected for the nontoxic AtnI_2_ and ppPLA_2_. Responses in response units (RU) were corrected for surface density of Bmh1 and Bmh2 and molecular weight of Bmh1, Bmh2 and sPLA_2_. The bars represent standard deviations of at least three independent measurements. AtxA – ammodytoxin A, AtxB – ammodytoxin B, AtxC – ammodytoxin C, bvPLA_2_– bee venom PLA_2_, bButx – β-bungarotoxin, AtnL – ammodytin L, Tpx – taipoxin, AtnI_2_– ammodytin I_2_, ppPLA_2_– porcine pancreatic PLA_2_.

It has been shown previously that both mammalian and yeast 14-3-3 proteins physically interact with AtxA, and proposed that AtxA inhibits the activity of Bmh1 and Bmh2 [14], [17]. Using surface plasmon resonance (SPR) we determined that other tested neurotoxic sPLA_2_s, bee venom sPLA_2_ (bvPLA_2_), β-bungarotoxin (bButx), ammodytoxin B (AtxB), ammodytoxin C (AtxC) and taipoxin (Tpx), but not the nontoxic ammodytin I_2_ (AtnI_2_) and porcine pancreatic sPLA_2_ (ppPLA_2_), can bind to Bmh1 and Bmh2 (Figure 5). AtnL, which is not neurotoxic but becomes inhibitory for yeast endocytosis upon the restoration of its PLA_2_ enzymatic activity (see above), can also bind to yeast 14-3-3 proteins. Binding was generally stronger to Bmh2 than to the 96% identical Bmh1.

Yeast 14-3-3 proteins have been found to localize to the plasma membrane [40]. To test whether they can be recruited there even without a physical interaction with another membrane associated protein, such as Yap1801, and whether their binding capacity depends on the membrane curvature, we determined the ability of 14-3-3 proteins to bind to phospholipid vesicles of different sizes. Using SPR, we measured the binding of recombinant purified Bmh1, Bmh2 and an equimolar mixture of both proteins, since 14-3-3 proteins can form functional heterodimers in addition to homodimers [34]. The average response of stably bound protein after 180 seconds of dissociation is shown in Table 1, and representative sensorgrams of the binding of Bmh1, Bmh2 and the Bmh1/Bmh2 mixture to phospholipid vesicles are presented in Supporting Information S1. We determined that Bmh1 and Bmh2, as well as the Bmh1/Bmh2 mixture, are able to bind directly to phospholipid vesicles. Bmh1 displayed a higher, but not statistically significant (average p-value 0.81 at the 95% confidence interval) binding than Bmh2 or the Bmh mixture. No significant differences between bindings to 50 nm, 100 nm or 200 nm vesicles were observed (Table 1). To confirm the direct interaction between the yeast 14-3-3 proteins and phospholipid vesicles, sedimentation experiments with Bmh2 were performed (Supporting Information S1). After 20 min incubation of the protein with POPC vesicles, at least 30% of the protein was detected in the retentate (lipid fraction) after centrifugation. Bmh2 protein can thus bind directly to phospholipid membranes and vesicles independently of membrane curvature.

**Table 1:** Binding of yeast 14-3-3 proteins to phospholipid vesicles.

	5 µM Bmh1		5 µM Bmh2		5 µM Bmh1+2	
	Av.	s.d.	Av.	s.d.	Av.	s.d.
**50 nm**	69.4	24.6	44.1	5.5	48.5	2.0
**100 nm**	66.7	25.1	47.3	16.4	47.2	0.9
**200 nm**	70.2	22.0	46.7	7.1	54.8	0.8

Surface plasmon resonance was used to analyze binding of 14-3-3 proteins to phospholipid vesicles. The response after 180 seconds of dissociation phase is shown. Av. – average response in response units (RU), s.d. – standard deviation in RU of at least three independent measurements.

## Discussion

Neurotoxic sPLA_2_s are components of animal venoms and their major pharmacological effect is inhibition of endocytosis. In this study we identified genetic interactions of a neurotoxic sPLA_2_ AtxA on a genome-wide level in the model organism budding yeast. Genes coding for proteins involved in endocytosis were enriched, and of the six genes with the highest confidence level three (*RVS161*, *YAP1802* and *BMH1*) have paralogs which are not in genetic interaction with AtxA. We show that in yeast AtxA inhibits the progression of endocytic vesicle formation, which does not result in disruption of the formation and distribution of sites of clathrin dependent endocytosis, but rather in changing vesicle formation dynamics. Exocytosis, on the other hand, is apparently not affected by AtxA since no significant changes in invertase secretion were observed (data not shown). Among the genetic interactors of AtxA, the most significant suppression of the effect of AtxA on the dynamics of endocytic vesicle formation was observed upon deletion of the *RVS161* gene, however, such suppression did not occur if the paralogous gene *RVS167* was deleted instead. Rvs161 and Rvs167 proteins are recruited to the endocytic vesicle site just before it is pinched off and they contain an N-BAR domain which senses, induces and stabilizes membrane curvature [41]. They have been shown to function as heterodimers in endocytosis [32], [33], and the stability of one isoform is diminished if the other is not present [32]. Our results indicate that the role of each isoform in endocytosis is however at least to a certain extent independent on the other isoform: additional expression of each protein individually stimulated endocytosis, whereas the inhibitory effect on endocytosis by AtxA is significantly more potent if Rvs161 is present, and Rvs167 cannot fully complement Rvs161 in this respect. Distinct behavior and functions of Rvs161/167 isoforms have been described previously. It has been shown that Rvs161 and Rvs167 display different sensitivities to latrunculin A treatment and different dependence upon F-actin for their localization at the plasma membrane [18]. Also, only Rvs161 has been shown to be required for cell fusion and this function was shown to be independent of its endocytic function [42]. Differences in the effect of AtxA on endocytosis depending on the presence and amount of Rvs161/167 proteins do not explain the observed specific genetic interaction of AtxA with *RVS161*, which could therefore also be independent of the endocytic function of Rvs161. Although interpretation of genetic interactions on the molecular mechanism level is difficult [43], in the case of *BMH1/2* and *YAP1801/1802* genes the results presented here and published previously are in agreement with the proposed explanation of the SDL data. While in this study we explained the relation between AtxA and Rvs161/167 in the context of endocytosis, which is important for understanding the molecular mechanism of the neurotoxicity of AtxA, other contexts of the interaction between AtxA and amphiphysin remain to be revealed.

It has previously been shown that sPLA_2_s affect the actin cytoskeleton when added to mammalian cells [28], [44], [45]. We could not observe any differences in the actin cytoskeleton between AtxA-expressing and control rhodamine-phalloidin stained cells (data not shown). On the other hand, the genetic interaction of AtxA with several actin cytoskeleton connected genes and its effect on Sac6-GFP patch lifetime point to a potential defect in bundling of actin filaments. This defect could explain the lack of internalization of the 6% of the patches, but not the whole extent of the reduction of endocytosis. The effect of AtxA on Sac6 and the partial recovery of the endocytic defect in the *yap1802Δ* and *bmh1/2Δ* strains indicate that the final endocytic defect caused by AtxA is a combination of several effects, among which however the modulation of Rvs161 function is the major contributor.

We addressed the question whether the reduction of endocytosis is dependent on enzymatic activity, and if other characteristics of AtxA may contribute to this effect. AtnL, the enzymatically inactive structural paralog of AtxA sharing 74% amino acid sequence identity [26] and capable of binding to 14-3-3 proteins, had no effect on endocytosis. Substitution of three residues in the calcium binding loop and one in the active site of AtnL renders the protein enzymatically active, while preserving its structural integrity [27]. The expression in yeast of two enzymatically active mutants (LV an LW) of AtnL, differing only in one residue, had an inhibitory effect on endocytosis implying that the observed effects of AtxA on endocytosis are caused by its enzymatic activity. Both mutants displayed a significantly lower effect than AtxA, which is in line with their one (LW) to two (LV) orders of magnitude lower enzymatic activity on PC-rich vesicles in comparison to AtxA, further confirming the importance of PLA_2_ enzymatic activity in impairment of endocytosis. Additionally, the slightly more potent effect on endocytosis observed in the case of LW mutant in comparison to LV is in accordance with its higher membrane binding affinity and activity particularly on PC-rich membranes [27]. Expression of the catalytically inactive H48Q mutant of AtxA had no effect on the phenotype of yeast cells (data not shown), but since neither this nor any other constructed mutant affecting the active site His-Asp diad of AtxA could fold correctly *in vitro* [46], we did not proceed working with these mutants. In agreement with previous studies on mammalian cells, which suggested the importance of binding to intracellular proteins for the neurotoxicity of sPLA_2_s [16], [17], [47], [48], we found that yeast 14-3-3 proteins are conserved enough for AtxA and other neurotoxic sPLA_2_s to bind to them. Moreover, deletion of *BMH1/2* genes suppressed the reduction of endocytosis by AtxA. Suppression of a similar extent was observed in the strain without the *YAP1802* gene, and based on this result and the published physical interaction between Bmh2 and Yap1801 [36] a possible interpretation of our experimental data is that AtxA binds to and inhibits the 14-3-3/AP180 complex. The observed effects of AtxA on endocytosis in yeast are therefore most likely the consequence of both localization to a specific part of the vesicle through protein-protein interactions and its enzymatic activity. To further elucidate the role of specific protein-protein interactions of AtxA, its *in vivo* co-localization with 14-3-3 and/or AP180 proteins at membrane invaginations should be examined.

On the basis of the data obtained in this study, the following mechanism for the reduction of endocytosis by AtxA can be proposed. In the first step in endocytosis, the vesicle coat proteins, including the adapter proteins Yap1801 and Yap1802, start to assemble, followed by 14-3-3 proteins that bind to both, the coat proteins through the physical interaction of Bmh2 and Yap1801 [36], and the phospholipid membrane directly. After this step, AtxA, in addition to non-specific interactions with intracellular membranes, can specifically bind to the nascent vesicle directly through binding to 14-3-3 proteins, which results in a localized higher-than-elsewhere PLA_2_ activity [49]. As the formation of the endocytic vesicle progresses, the curvature of the vesicle membrane increases, promoting the enzymatic activity of AtxA (Figure 4). The resulting faster phospholipid hydrolysis in turn promotes even more positive membrane curvature [11], [12]. This interferes with the activity of the membrane curvature sensing amphiphysins (especially Rvs161) and consequently the dynamics of vesicle formation and scission. The inhibition is manifested in a longer before inward movement part of the lifetime of Sla1 and doubled Rvs161-GFP patch lifetime.

All of the proteins that are involved in the formation and scission of the vesicle from the plasma membrane are conserved from yeast to mammals [50], [51], therefore the proposed mechanism of action of AtxA can be translated from yeast cells to the presynaptic cell of neuro-muscular junctions, where AtxA inhibits the release of the neurotransmitter acetylcholine into the synaptic cleft. For example, the mammalian coat protein AP180, whose homologs in yeast are Yap1801 and Yap1802, is a neuronal-specific adaptor protein involved in the endocytic recycling of synaptic vesicles that binds clathrin and promotes its assembly into cages [52]–[56]. In mammalian cells, the morphological effects accordant with the reduction of endocytosis by sPLA_2_s – Ω-shaped invaginations at the plasma membrane and reduced number of synaptic vesicles – have been observed when these neurotoxic enzymes were added extracellularly [8]–[10], [13], [57]. It has been shown that AtxA can internalize within minutes into mouse motoneuron-like cells *in vitro* [28], and into mammalian motor nerve terminals *in vivo* [58]. Intracellular activity of AtxA in mammalian cells is therefore also likely and the yeast model presented here suggests that AtxA, by reducing endocytosis after binding to a specific region of the endocytic vesicle and changing membrane properties through the products of its hydrolysis, could affect synaptic vesicle recycling and reduce the number of synaptic vesicles and released neurotransmitter molecules. AtxA has been found to bind to 14-3-3 proteins [17], whose homologs in yeast, Bmh1 and Bmh2, have been shown to localize to the plasma membrane enriched fraction [41]. Here we show that both Bmh proteins are capable of direct binding to phospholipid bilayers independently of membrane curvature, and a similar phospholipid binding ability has been shown for mammalian 14-3-3 proteins [59], [60]. This binding could aid in the specific localization of AtxA at the site of vesicle formation early in the process, when membrane curvature is not yet the driving force for the recruitment of AtxA. Moreover, the interaction with 14-3-3 proteins is shown here for different neurotoxic sPLA_2_s. One major difference between clathrin dependent endocytosis in yeast and mammalian cells however is in the relative contributions of the actin cytoskeleton, clathrin, amphiphysin and dynamin [61], [62]. In mammalian cells, clathrin and dynamin are essential [61], [63], [64], whereas in yeast they play only a minor role. On the other hand, the actin cytoskeleton is essential for vesicle formation in yeast cells [18], [25], [65] while in mammalian cells it only plays a minor role. According to the proposed mechanism, interference with amphiphysin Rvs161 activity through modulation of membrane curvature is crucial for the effect of AtxA on endocytosis. In mammalian cells, amphiphysin stabilizes the neck of clathrin coated synaptic vesicles [66], [67] and is needed for recruitment of the pinchase dynamin to the forming vesicle [68], [69]. It has been proposed that, similar to yeast, membrane curvature orchestrates the recruitment of proteins and progression of vesicle formation in mammalian cells [70]. This is in accordance with the observation that during vesicle squeezing membrane curvature deviates from the optimal dynamin binding curvature which leads to dissociation of dynamin from the vesicle before the actual scission [71], [72]. AtxA, and likely also other neurotoxic sPLA_2_s, with their intracellular enzymatic activity that changes membrane curvature thus affect conserved components of endocytosis that are needed for vesicle scission from the plasma membrane and depend on membrane curvature for their proper activity both in yeast and in mammalian cells. Further studies will however be necessary to identify the major molecular targets of AtxA and other sPLA_2_s in mammalian neuronal cells.

## Materials and Methods

### Yeast Strains and Plasmids

Yeast strains used in this study for microscopy experiments were DDY2734, DDY3069, DDY3070, DDY3096, DDY3097, DDY3118, DDY3100 [18], DDY*yap1802Δ* (*his3-Δ200 ura3–52 leu2–3,112 SLA1-GFP::HIS3 yap1802Δ::kanMX*), *bmh1Δ* (*his3-Δ200 ura3–52 leu2–3,112 SLA1-GFP::natMX bmh1Δ::kanMX*), *bmh2Δ* (*his3-Δ200 ura3–52 leu2–3,112 SLA1-GFP::natMX bmh2Δ::kanMX*). For LY internalization experiments the strains used were BY4741, *rvs161Δ* and *rvs167Δ* (Euroscarf), BY4049, BY4063 and BY4395 [73] and for the SDL screen MMY1001 [74]. Additionally, for microscopy experiments some of the above mentioned yeast strains carrying the *GAL1pr-ATXA natMX* cassette at the *MFA1* locus used were constructed as described [74]. Plasmids used in this study in the LY internalization experiments were pRD53 [75], pYUP1.3 [14], pYUP2.1 (pYUP1.3 with *LEU2* instead of *URA3* marker) and pGalL (empty pYUP2.1). For recombinant yeast 14-3-3 expression, plasmids pBM1.3 (for Bmh1 expression) and pBM2.4 (for Bmh2 expression) were used. Both plasmids were constructed by PCR amplification of the *BMH1* or *BMH2* genes from yeast genomic DNA and its insertion between *Nde*I and *Cla*I restriction sites of plasmid pT7–7 [76]. Plamids pCRGA (expressing AtxA), pCRGL (expressing AtnL), pCRGLV (expressing AtnL-H28Y/L31V/N33G/S49D  =  LV) and pCRGLW (expressing AtnL-H28Y/L31W/N33G/S49D  =  LW) used in microscopy experiments were constructed by inserting the genes for *AtxA*, *AtnL*, or the LV and LW mutants of AtnL [27] between *Bgl*II and *Cla*I sites of plasmid pCRGU that was constructed by inserting monomeric RFP into pCGGU [77], [78]. For additional *RVS* expression in the DDY *rvs* deletion strains carrying the *GAL1pr-ATXA natMX* cassette at the *MFA1* locus, plasmids expressing either *RVS161* or *RVS167* under their native promoters and corresponding empty plasmid from the MoBY-ORF collection were used [79]. Unless otherwise stated, yeast strains were grown in appropriate synthetic drop-out media for plasmid selection or in YPD media at 30°C. Growth of strains for microscopy experiments is described below.

### Systematic Identification of the Genetic Interactors of AtxA

The detailed protocol for synthetic dosage lethality (SDL) screens was described previously [74]. Briefly, the yeast strain carrying the *GAL1pr-ATXA natMX* cassette in the *MFA1* locus was crossed to the non essential gene deletion collection and double mutant haploids were selected and tested for growth rate on glucose and galactose. To identify the genetic interactors in the quantitative SDL screen, the threshold was set such that a relative growth fitness lower than 0.4 in one biological repetition and lower than 0.67 in the other biological repetition was required, or alternatively a relative growth fitness below 0.6 was required in both biological replicates. In the visually inspected screen, the genetic interactors were identified as having an obvious growth defect in at least one of the biological replicates.

### Phospholipids and Vesicle Preparation

POPC (1-palmitoyl-2-oleoyl-*sn*-glycero-3-phosphocho-line), DOPC (1,2-dioleoyl-*sn*-glycero-3-phosphocholine) and DPPS (1,2-dipalmitoyl-*sn*-glycero-3-phospho-L-serine) were from Avanti Polar Lipids, USA. Unilamellar vesicles were prepared in appropriate buffer by extrusion [80] through polycarbonate membranes with pore diameters of 50 nm, 100 nm, or 200 nm (Nucleopore Track-Etch Membrane PC, Whatman, USA). The actual size of the vesicles was determined using dynamic light scattering method and the measurements are represented in Supporting Information S1.

### PLA_2_ Enzymatic Activity Measurement

The initial rate of hydrolysis of phospholipids by AtxA was measured by monitoring the displacement of a fluorescent fatty acid analogue (11-dansylundecanoic acid; Molecular Probes, USA) from fatty acid-binding protein as described [49], [81]. Assays were performed in Hanks’ balanced salt solution with 0.9 mM Ca^2+^ and 1.27 mM Mg^2+^ (Invitrogen, USA) containing 30 µM POPC vesicles (50 nm, 100 nm or 200 nm diameter), 1 µM 11-dansylundecanoic acid and 10 µg recombinant fatty acid-binding protein. Solutions with a final volume of 1.3 ml were assayed in acrylic fluorometric cuvettes at 37°C with magnetic stirring, using a Perkin-Elmer LS50B spectrofluorometer. Excitation was at 350 nm and emission at 500 nm, with 10 nm slit widths. Reactions were started by adding 10 ng AtxA, typically in 1 µl, which resulted in a slope of approximately 45 degrees. Three independent replicates were performed. All dilutions were prepared in buffer containing 1 mg/ml fatty acid-free BSA (Sigma) to prevent loss of enzyme due to adsorption to the walls of the tube.

### Confocal Microscopy

To determine the average number of endocytic sites per yeast cell, Sla1-GFP patches in wild-type and AtxA-expressing strains were analyzed using confocal microscopy. Cells were grown to early logarithmic phase in raffinose containing minimal media at 25°C, washed with water and grown in galactose containing minimal media for 6 h to induce AtxA expression. The cell sample was prepared on a thin agar layer on standard glass slide. Microscopy was performed using a Leica TCS SP2 confocal microscope (Leica Microsystems, Germany). A 100x oil immersion objective (HCX PLAPO CS, NA: 1.40) was used. GFP fluorescence was excited at 488 nm and emission recorded at 500–550 nm. Approximately 40 optical sections with 0.12 µm thickness per sample were imaged. Leica Confocal software was used for microscope control and image acquisition. Deconvolution of acquired image stacks was done with Huygens Professional (Scientific Volume Imaging, the Netherlands). Image processing and 3D reconstruction were done with Amira 4 (Mercury Computer Systems, USA) and LAS AF Lite (Leica Microsystems, Germany) software. Sites of endocytosis were counted both manually and automatically from overlaid images of maximum intensity projections of the GFP signal and DIC images. At least 100 cells per sample were analyzed.

### Real-time Fluorescence Microscopy

Real time fluorescence microscopy was used to monitor the dynamics of different GFP tagged endocytic proteins in strains expressing sPLA_2_ and corresponding control strains. Either the effect of AtxA expression from a single copy of its gene inserted in the genome (*mfa1Δ::GAL1_pr_-ATXA::natMX*), prepared as described in [74], or the effects of AtxA, AtnL and its enzymatically active mutants expressed from plasmid clones were monitored. When expressed from plasmid, the sPLA_2_ were C-terminally tagged with mRFP to monitor their presence in the cell. Yeast cells were grown as described above. Cells were attached to concanavalin A-coated coverslips, which were sealed to standard glass slides with vacuum grease (Dow Corning). An inverted Olympus IX81 microscope equipped with 100x/NA 1.4 immersion objective and Orca-II CCD camera (Hamamatsu, Japan) was used. All images were acquired at 25°C as described [18], [25]. The Metamorph 7.1 software (Molecular Devices) was used to control the microscope and acquire images. Images were acquired in continuous mode for 2–4 minutes at a rate of 1 frame/second for Sla1-GFP and 4 frames/second for Rvs161-GFP, Rvs167-GFP and Sac6-GFP. Image analysis – background subtraction and photo bleaching correction – was performed with ImageJ, as described [25]. At least 100 patches from several cells were analyzed per strain unless otherwise noted. In samples expressing sPLA_2_-mRFP fusion proteins only the cells with detectable RFP signal were analyzed. The Particle tracking algorithm was written in OpenCV. To assess the statistical significance of the effect of AtxA expression on patch lifetimes compared to the corresponding control strains, p-values at a 95% confidence interval were calculated using the *t*-test.

### Recombinant Yeast 14-3-3 Protein Expression and Purification

Recombinant Bmh1 and Bmh2 were expressed in BL21(DE3) *E. coli* cells. The expression from plasmids pBM1.3 (*BMH1* coding) and pBM2.4 (*BMH2* coding) was induced with 0.1 mM IPTG for 5 h at 37°C. Cells were lysed in buffer A (0.25 M sucrose, 20 mM Tris/HCl, pH 7.5, 2 mM EDTA, 10 mM EGTA and 1 mM dithiothreitol) containing protease inhibitor cocktail using sonication. Bmh1 and Bmh2 were purified from cell lysates as described [82]. Western blot analysis, using rabbit anti-14-3-3*β* polyclonal antibodies (Santa Cruz Biotechnology, USA) and N-terminal amino acid sequencing were performed to verify the identity of the purified recombinant 14-3-3 proteins. Moreover, the purified recombinant Bmh proteins were able to form dimers, as determined with dynamic light scattering measurements.

### Surface Plasmon Resonance Measurements

Surface plasmon resonance (SPR) experiments were performed at 25°C using a BiacoreX system (Biacore AB, Sweden). BIAevaluation software was used for measurement analysis. For the analysis of binding of sPLA_2_s to recombinant yeast 14-3-3 proteins, recombinant Bmh1 and Bmh2 proteins in 50 mM Tris (pH 7.5), 150 mM NaCl and 0.1 mM CaCl_2_ were immobilized to CM5 sensor chips. 2 µM solutions of sPLA_2_ (neurotoxic AtxA, AtxB, AtxC, β-bungarotoxin, taipoxin and bee venom PLA_2_, non-neurotoxic AtnL and AtnI_2_, and porcine pancreatic PLA_2_) were injected over the chip surface. The dissociation was monitored for 180 seconds. The sensor chip was regenerated with 5 mM NaOH between consecutive injections. Sensorgrams were corrected to account for the differences in molecular weight of different PLA_2_s, the amount of Bmh immobilized on the chip and the differences in molecular weight of Bmh by the following equation, adapted from [83]:
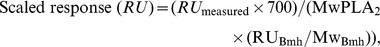
where RU_measured_ is the signal measured during the test, MwPLA_2_ is the molecular weight of PLA_2_, RU_Bmh_ is the amount of Bmh immobilized on the chip, and Mw_Bmh_ is the molecular weight of Bmh.

For the analysis of binding of recombinant yeast 14-3-3 proteins to phospholipid vesicle, 75% DOPC/25% DPPS (mol/mol) vesicles of different diameters (50 nm, 100 nm in 200 nm) were used. All measurements were performed at 25°C. The working buffer (140 mM NaCl, 20 mM NaH_2_PO_4_, 1 mM EDTA; pH 7.5) was degassed and filtered through a 0.22 µm filter prior to use. The L1 chip was prepared as described [27], [84]. 5 µM solutions of Bmh1, Bmh2 or an equimolar mixture of both were injected over the chip surface at 40 µl/min. The running buffer contained 1% BSA in order to prevent non-specific interaction of the Bmh1/2 proteins with the sensor chip surface. The dissociation was monitored for 180 seconds.

### Endocytosis Assay

The uptake rate of the fluid-phase endocytosis marker Lucifer Yellow (Lucifer Yellow CH dilithium salt, Sigma) in wild-type, *rvs161Δ*, *rvs167Δ* and *rvs161Δ rvs167Δ* strains expressing AtxA and corresponding control strains was quantified as described [24], [85]. Briefly, cells were grown to mid-logarithmic phase, harvested and resuspended in fresh medium containing 4 mg/ml LY and 20 mM NaN_3_ to prevent cell growth during LY staining. After 1 h incubation at 30°C or 0°C, cells were washed eight times with ice-cold buffer A (50 mM Na-succinate, pH 5.0, 100 mM NaCl, 10 mM MgCl_2_, 20 mM NaN_3_) containing 10% sorbitol to prevent lysis of more fragile cells. The cells were collected by centrifugation. They were then resuspended in 1 ml buffer B (50 mM Tris-HCl, pH 7.5, 10 mM 2-mercaptoethanol) and 100 U lyticase was added. After 1 h incubation at 37°C (complete lysis), 50 µl of 10% SDS was added to the lysed cells. LY fluorescence was excited at 426 nm and emission measured at 550 nm, both with 15 nm slit widths, using a Perkin Elmer LS50B spectrofluorometer. To assess the statistical significance of the determined differences in the uptake rates of LY in AtxA-expressing strains compared to the corresponding control strains, p-values at a 95% confidence interval were calculated using the *t*-test.

## Supporting Information

Supporting Information S1 (DOCX)
