# Supplementary material for: A Neurotoxic Phospholipase A2 Impairs Yeast Amphiphysin Activity and Reduces Endocytosis
Source: PLoS One. 2012 Jul 23;7(7):e40931. doi: 10.1371/journal.pone.0040931 (PMC3402474; doi:10.1371/journal.pone.0040931)
Supplement: Supporting Information S1 — (DOCX) [file pone.0040931.s001.docx]

**Supporting Information S1**

**Supplemental Materials and Methods**

*Dynamic light scattering measurements*

POPC vesicles were prepared by extrusion through polycarbonate membranes with pore sizes of 30, 50, 100 and 200 nm as described in the Materials and Methods section. Dynamic light scattering measurements were performed at 37 °C using a PDDLS/BatchPlus System (Precision Detectors, USA). Assay solutions with a final volume of 400 *µ*L containing 30 *μ*M phospholipid were prepared in Hanks’ Balanced Salt Solution with 1.26 mm Ca^2+^. Sixty accumulated correlation functions, obtained with a run time of 10 s and sample time set to 10 *μ*s, were used per measurement to calculate the diameter of scattering particles using a smoothness factor of 20 with the PrecisionDeconvolve software provided by the manufacturer. Representative size distribution plots are shown in Figure S1.

*Sedimentation experiment*

The Bmh sedimentation experiment was performed as described previously [1] with some modifications. Six µg of Bmh2 were incubated with phospholipid vesicles (lipid/protein ratio 1000 (mol/mol)) in 900 µl SPR working buffer (composition described in the Materials and Methods section) for 20 minutes at room temperature. After the incubation, the sample was loaded onto Centricon YM-100 (Amicon) and centrifuged at 1000 g for 30 min at 4 °C. Proteins present in the retentate and filtrate were precipitated with 10 % TCA, loaded on a SDS-PAGE gel and stained with Coomassie brilliant blue after electrophoresis. The gel was scanned and the average pixel densities of Bmh2 protein bands of 31 kDa were background corrected and quantified using ImageJ software (National Institute of Health).

**Supplemental References**

1. Podlesnik Beseničar M, Metkar S, Wang B, Froelich CF, Anderluh G (2008) Granzyme B translocates across the lipid membrane only in the presence of lytic agents. Biochem Biophys Res Commun 371: 391-394.

**Supplemental Figures**

Figure S1


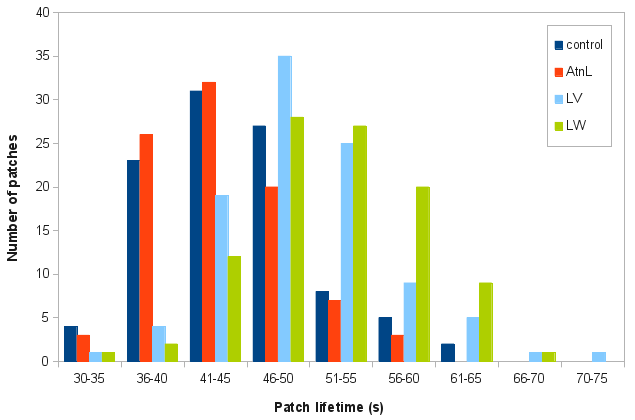


**Figure S1. Distribution of Sla1-GFP patch lifetimes in yeast cells expressing AtnL, the LV and LW mutant of AtnL, and in control cells.**

The lifetimes of 100 Sla1-GFP patches from control yeast cells (dark blue), cells expressing AtnL (red), and the LV (cyan) or LW (green) AtnL mutant were analyzed. Cells expressing the LW mutant have significantly higher number of patches with lifetimes longer than 55 seconds than the LV mutant.

Figure S2


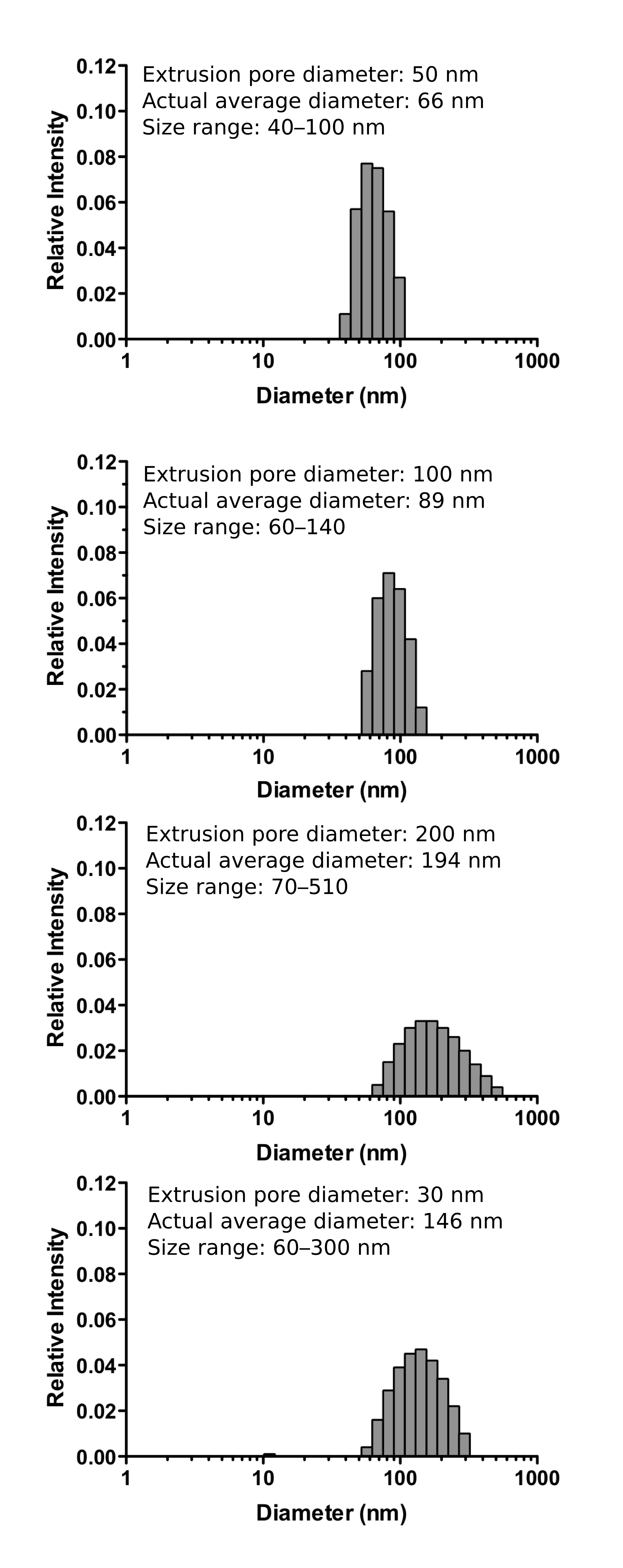


**Figure S2. Size Distribution of Extruded POPC Vesicles Determined by Dynamic Light Scattering.**

The average diameters of POPC vesicles used in this study correlated very well with their expected diameter sizes of 50, 100 and 200 nm. The determined average vesicle diameters in a typical measurement were 66, 89 and 194 nm, respectively. The sizes of individual particles in these vesicle populations were in the range of 40–100 nm, 60–140 nm, and 70–510 nm, respectively. On the contrary, our measurements indicate that POPC vesicles prepared by extrusion through 30 nm pores fuse into larger particles with an average diameter of 146 nm and a broad size distribution in the range 60–300 nm.

Figure S3


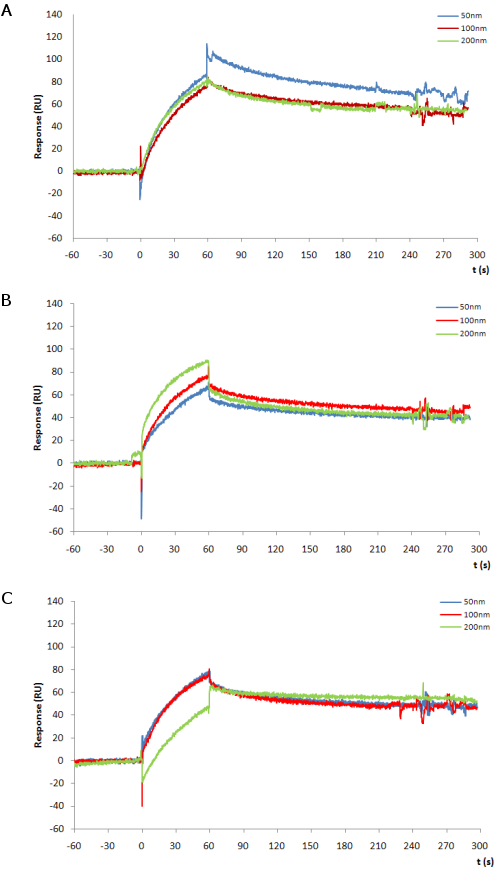


**Figure S3. Yeast 14-3-3 proteins bind directly to phospholipid membranes.**

Representative binding of yeast 14-3-3 proteins to vesicles of different diameters. (A) Binding of Bmh1. (B) Binding of Bmh2. (C) Binding of the equimolar Bmh1/Bmh2 mixture. 50 nm vesicles - blue line, 100 nm vesicles - red line, 200 nm vesicles - green line.
